# Angiotensin II Receptor Blocker Associated With Less Outcome Risk in Patients With Acute Kidney Disease

**DOI:** 10.3389/fphar.2022.714658

**Published:** 2022-04-20

**Authors:** Vin-Cent Wu, Yu-Feng Lin, Nai-Chi Teng, Shao-Yu Yang, Nai-Kuan Chou, Chun-Hao Tsao, Yung-Ming Chen, Jeff S Chueh, Likwang Chen

**Affiliations:** ^1^ Department of Internal Medicine, National Taiwan University Hospital, Taipei, Taiwan; ^2^ Institute of Population Health Sciences, National Health Research Institutes, Miaoli, Taiwan; ^3^ Department of Surgery, National Taiwan University Hospital, Taipei, Taiwan; ^4^ Glickman Urological and Kidney Institute, Cleveland Clinic Lerner College of Medicine, Cleveland Clinic, Cleveland, OH, United States; ^5^ Department of Urology, National Taiwan University Hospital, Taipei, Taiwan

**Keywords:** ARB, ACEi, AKD, AKI, dialysis, weaning, mortality, hyperkalemia

## Abstract

**Objective:** The aim of this study was to explore the respective use of angiotensin-converting-enzyme inhibitors (ACEis) or angiotensin receptor blockers (ARBs) on the outcomes of patients who could be weaned from dialysis-requiring acute kidney injury (AKI-D).

**Methods:** This case–control study enrolled 41,731 patients who were weaned from AKI-D for at least 7 days from Taiwan’s National Health Insurance Administration. We further grouped AKI-D patients according to ACEi and ARB use to evaluate subsequent risks of all-cause mortality and re-dialysis. The outcomes included the all-cause mortality and new-onset of end-stage kidney disease (ESKD; re-dialysis) following withdraw from AKI-D.

**Results:** A total of 17,141 (41.1%) patients surviving AKI-D could be weaned from dialysis for at least 7 days. The overall events of mortality were 366 (48.9%) in ACEi users, 659 (52.1%) in ARB users, and 6,261 (41.3%) in ACEi/ARB nonusers, during a mean follow-up period of 1.01 years after weaning from AKI-D. In regard to all-cause of mortality, pre-dialysis ARB users had lower incidence than ACEi users [hazard ratio (HR 0.82), *p* = 0.017]. Compared with ACEi/ARB nonusers, continuing ARB users had a significantly low risk of long-term all-cause mortality (adjusted hazard ratio 0.51, *p* = 0.013) after propensity score matching. However, new users of ACEi at the acute kidney disease (AKD) period had a higher risk of re-dialysis after weaning than ACEi/ARB nonusers (aHR 1.82, *p* < 0.001), whereas neither ACEi nor ARB users confronted significantly increased risks of hyperkalemia after weaning.

**Conclusions:** Compared with patients without ACEi/ARB, those continuing to use ARB before the event and after weaning had low all-cause mortality, while new users of ACEi at AKD had increased risk of re-dialysis. AKI-D patients continuing to use ACEi or ARB did not have higher risk of hyperkalemia. Future prospective randomized trials are expected to confirm these findings.

## Introduction

Angiotensin-converting-enzyme inhibitors (ACEis) and angiotensin receptor blockers (ARBs) are both frequently prescribed in treating patients with hypertension, congestive heart failure, or chronic kidney disease. However, due to the concern of their possible worsening effect on acute kidney function impairment, it has been suggested that ACEi/ARB should be withheld prior to or during some clinical scenarios (Che et al., 2011). The Kidney Disease: Improving Global Outcomes (KDIGO) Conference developed a consensus that ACEi/ARB should be temporarily discontinued during acute kidney injury (AKI) and reinitiated in acute kidney disease (AKD) (Ostermann et al., 2020a; Ostermann et al., 2020b). The consensus report of the Acute Disease Quality Initiative (ADQI) 22 workshop further proposed the recommendations regarding the ACEi/ARB treatment of patients in AKD (Liu et al., 2020). However, the impact of either ACEi or ARB alone on kidney function of patients who were weaned-off from dialysis-requiring AKI (AKI-D) has not been well discussed.

Previous studies explored the impacts of the use of ACEi/ARB on post-AKI patients provided conflicting data (Arora et al., 2008; Coca et al., 2013; Yang et al., 2020). ACEi and ARB are distinctive because they suppress different parts of the renin–angiotensin–aldosterone system (RAS), even though many of their pharmacologic effects could be similar. Therefore, focused exploration on the specific effects of ACEi versus ARB may help to clarify their respective effects on important outcomes in various clinical scenarios, considering their potentially different effects on RAS components (Vaduganathan et al., 2020).

In a study of 14,117 patients with pre-dialysis stage 5 chronic kidney disease (CKD), ACEi users were associated with higher mortality rates than ARB users, particularly in a subgroup of diabetic patients (Lin et al., 2017). Currently, there is no large-scale study addressing the specific role of ACEi or ARB supremacy regarding the long-term mortality and/or future dialysis-dependence (re-dialysis) in AKI-D patients who were successfully weaned-off from dialysis. Previous studies examining the effects of these two kinds of agents on postoperative AKI rarely explored their respective roles. In patients with AKD, the superiority of ARB over ACE inhibitors in regard to all-cause mortality and long-term kidney function remains to be proven. Therefore, in this study, we aimed to explore whether the respective use of ACEi or ARB is related to long-term mortality and/or eventual development of end-stage kidney disease (ESKD) in patients who were weaned-off from AKI-D.

## Methods

### Patients

The study enrolled all patients between 18 and 80 years of age who were diagnosed with AKI and underwent dialysis treatment from May 2015 to December 2017 in Taiwan, with the final patient follow-up on 31 December 2018 (i.e., at least 1 year follow-up). Our study used a longitudinal database through the Applied Health Research Data Integration Service from Taiwan’s National Health Insurance Administration (NHIA). The longitudinal database contains comprehensive healthcare information, including but not limited to data regarding individual demographic background, acute inpatient hospitals, outpatient primary care and subspecialty office visits, outpatient pharmacies, diagnoses, prescriptions, long-term care facilities, and medical events. To detect possible fraud in the NHI, the NHIA has been routinely auditing data and records submitted by healthcare institutions and providers (Wu et al., 2015). The NHIA is the only insurance carrier of covered healthcare in Taiwan. To avoid rejection of claim reimbursement from the NHIA, physicians in Taiwan usually follow clinical guidelines/policy suggested by the consensus. This study excluded patients who had undergone nephrectomy, chronic dialysis, or renal transplantation before the index date.

The study protocol was approved by the Research Ethics Committee of the National Taiwan University Hospital (201807119RIND) and that of National Health Research Institutes (EC1060402-E), and the need for informed consent was waived because of its retrospective nature and no identifiable patient data could be accessible.

### The Use of ACEi/ARB Before AKI-D

**FIGURE 1:**
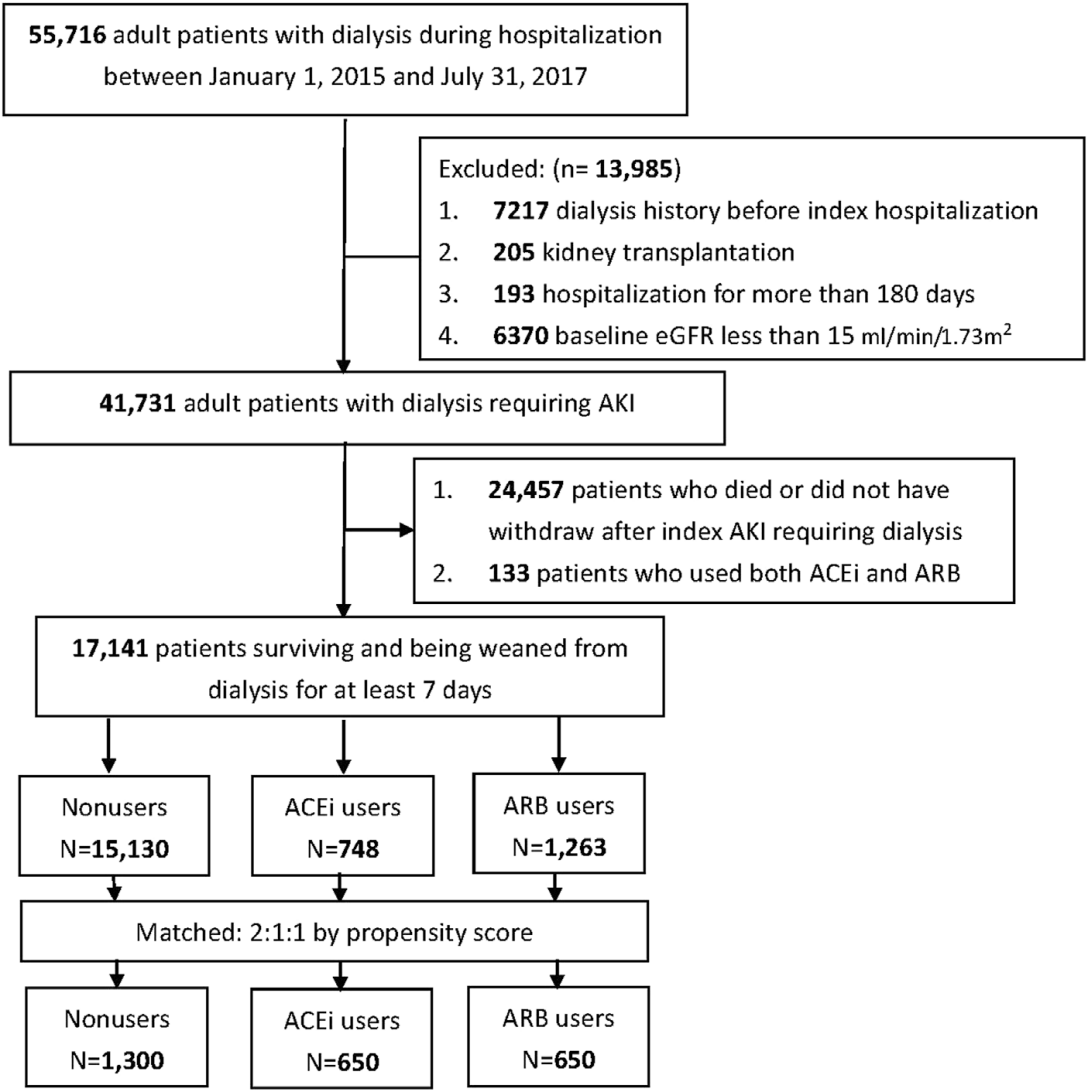
Flow diagram of the enrollee.

The records of taking ACEi or ARB within 180 days before index dialysis were enrolled to determine the grouping of the patients. The patients who were treated simultaneously with both ACEi and ARB within 180 days before index dialysis were excluded to avoid misclassification (*n* = 133), and the others were classified into following three groups before dialysis: AKI-D patients who were administered ACEi (ACEi users), who were administered ARB (ARB users), and who were administered neither ACEi nor ARB (ACEi/ARB nonusers). The baseline demographic data, comorbidities, and the prescribed medications of these patients were collected (Figure 1). The estimated glomerular filtration rate (eGFR) was calculated according to the Modification of Diet in Renal Disease Study equation according to the baseline serum creatinine (sCr). Baseline sCr was the nadir value obtained after the previous admission in those who had more than one admission within 6 months before the index admission or the mean outpatient sCr level in those without previous admission within 180 days before the index admission (Shu et al., 2016).

### The Measurement of ACEi/ARB Exposure After Weaning From AKI-D

The use of the RAS inhibitors at weaning from AKI-D was defined as the use of a RASi during 90 days after withdrawal from dialysis (AKD-period). We further inspected the respective effects of RASi during the AKD period. The patients who were not administered RAS inhibitors during AKD were divided into following three groups: prior ACEi user (prior ACEi usage only), prior ARB user (prior ARB usage only), and ACEi/ARB nonusers (neither ACEI nor ARB usage), while the patients who kept on usage of RASi were divided into continuing ARB and continuing ACEi users. The new user of RAS inhibitors during the AKD periods were defined as new users.

### Comorbidities

For analysis of comorbid conditions of these patients, we calculated the Charlson comorbidity index scores (Charlson et al., 1987) by coding from the International Classification of Disease, 9^th^ Revision, Clinical Modification (ICD-9-CM), and ICD-10 administrative data (Quan et al., 2005). Gastrointestinal bleeding was ascertained from emergency department visits and hospitalization episodes using ICD-9 diagnosis codes.

### Outcomes of Interest

The main outcomes included the all-cause mortality and new-onset of end-stage kidney disease (ESKD; re-dialysis) following withdrawal from AKI-D. Additional outcome measures included hospitalization with a major adverse cardiovascular event (MACE) as the major diagnosis and MACE-related death, which was defined as death with hospitalization for MACE during the 90 days prior to the death.

Finally, to assess whether observed associations between the respective ACEi or ARB administration and the outcomes of interest were attributable to different health statuses, we further compared the risk of gastrointestinal bleeding, an outcome believed not to be affected by the choice of RASi treatments, as a negative control test.

### Statistical Analysis

The baseline variables were shown as means ± standard deviations (SDs) for continuous variables and percentages for categorical variables in the three groups. Differences between these groups were compared using the *post hoc* analysis consisting of t-tests with the Bonferroni correction for continuous variables and the χ^2^ test for categorical variables. We compared the risks for AKI and mortality among these aforementioned groups using the COX proportional hazard model, adjusting for age, gender, medication, comorbidities, and eGFR (Table 1). The significance levels for entry (SLE) and for stay (SLS) were set to 0.15 to be conservative. Consequently, with the aid of substantive knowledge, the best candidate final COX proportional model was identified manually by dropping the covariates with *p* value >0.05 one at a time until all regression coefficients were significantly different from 0. Because of the high mortality rate in patients after AKD, competing risk regression analysis taking mortality into consideration was also performed using the Fine and Gray model to calculate the sub-distribution hazard (sHR) (Austin et al., 2016).

**TABLE 1:** Comparison of patient baseline characteristics among ACEi users, ARB users, and other drug users.

Characteristic	Before match				Propensity score-matched			
	ACEi user (*n* = 748)	ARB user (*n* = 1,263)	Nonuser (*n* = 15,130)	*p*-value	ACEi user (*n* = 650)	ARB user (*n* = 650)	Nonuser (*n* = 1,300)	*p*-value^1^
Age (year)				<0.001	—	—	—	0.945
18–54	102 (13.6)	151 (12.0)	3110 (20.6)		85 (13.1)	85 (13.1)	155 (11.9)	
55–64	154 (20.5)	230 (18.2)	2825 (18.6)		136 (20.9)	135 (20.8)	275 (21.2)	
65–74	196 (26.2)	336 (26.6)	3264 (21.5)		176 (27.1)	166 (25.5)	328 (25.2)	
75–84	204 (27.2)	383 (30.3)	3968 (26.2)		171 (26.3)	185 (28.5)	382 (29.4)	
85+	92 (12.2)	163 (12.9)	1963 (12.9)		82 (12.6)	79 (12.2)	160 (12.3)	
Insured salary, $								
<814.3	255 (34.0)	416 (32.9)	5633 (37.2)	0.015	224 (34.5)	220 (33.8)	446 (34.3)	0.579
814.3–1635.7	427 (57.0)	724 (57.3)	8095 (53.5)		373 (57.4)	377 (58.0)	770 (8.2)	
>1635.7	66 (8.8)	123 (9.7)	1402 (9.2)		53 (8.2)	53 (8.2)	84 (6.5)	
Male	434 (58.0)	705 (55.8)	8931 (59.0)	0.077	383 (58.9)	379 (58.3)	772 (59.4)	0.900
No. of hospitalization (/3 years)	3.39 ± 4.02	2.75 ± 3.10	2.70 ± 3.55	<0.001	3.49 ± 4.12	2.48 ± 2.73	3.11 ± 3.63	<0.001
Baseline comorbidities								
Charlson score				<0.001	—	—	—	0.826
0	308 (41.1)	210 (16.6)	8623 (56.9)		213 (32.8)	210 (32.3)	449 (34.5)	
1–2	148 (19.7)	320 (25.3)	2684 (17.7)		147 (22.6)	152 (23.4)	314 (24.2)	
3–4	159 (21.2)	343 (27.1)	2096 (13.8)		157 (24.2)	159 (24.5)	291 (22.4)	
≥5	133 (17.7)	390 (30.8)	1727 (11.4)		133 (20.5)	129 (19.9)	246 (18.9)	
Antihypertension drugs								
Alpha-blocker	39 (5.2)	116 (9.2)	339 (2.2)	<0.001	35 (5.4)	45 (6.9)	40 (3.1)	<0.001
Beta-blocker	214 (28.6)	501 (39.7)	1797 (11.9)	<0.001	191 (29.4)	230 (35.4)	220 (16.9)	<0.001
Ca-blocker	276 (36.9)	838 (66.4)	2360 (15.6)	<0.001	248 (38.2)	417 (64.2)	276 (21.2)	<0.001
Diuretics	51 (6.8)	127 (10.1)	487 (3.22)	<0.001	48 (7.4)	61 (9.4)	46 (3.5)	<0.001
others	41 (5.5)	98 (7.8)	314 (2.1)	<0.001	38 (5.9)	33 (5.1)	45 (3.5)	0.0378
Peptic ulcer disease	97 (12.9)	196 (15.5)	1101 (7.2)	<0.001	97 (14.9)	68 (10.5)	134 (10.3)	0.006
Mild liver disease	36 (4.8)	92 (7.2)	569 (3.7)	<0.001	35 (5.4)	38 (5.9)	58 (4.5)	0.376
DM	119 (15.9)	388 (30.7)	1619 (10.7)	<0.001	119 (18.3)	147 (22.6)	256 (19.7)	0.135
HTN	615 (82.2)	1096 (86.8)	9493 (62.7)	<0.001	529 (81.4)	553 (85.1)	1120 (86.2)	0.021
Myocardial infarction	37 (4.9)	69 (5.4)	231 (1.5)	<0.001	36 (5.5)	24 (3.7)	35 (2.7)	0.006
Peripheral vascular disease	21 (2.8)	57 (4.5)	203 (1.3)	<0.001	20 (3.1)	28 (4.3)	38 (2.9)	0.253
Cerebrovascular disease	72 (9.6)	194 (15.3)	917 (6.0)	<0.001	72 (11.1)	78 (12.0)	150 (11.5)	0.873
Dementia	31 (4.1)	65 (5.1)	426 (2.8)	<0.001	31 (4.8)	25 (3.9)	59 (4.5)	0.691
COPD	73 (9.7)	191 (15.1)	1009 (6.6)	<0.001	73 (11.2)	78 (12.0)	127 (9.8)	0.283
Gout	261 (34.8)	453 (35.8)	4570 (30.2)	<0.001	212 (32.6)	224 (34.5)	449 (34.5)	0.676
Baseline eGFR ( ml/min/1.73^2^)	44.5 ± 37.7	38.8 ± 41.5	50.1 ± 48.1	<0.001	44.5 ± 36.4	43.6 ± 45.5	44.3 ± 45.2	0.915
CKD stage				<0.001				0.854
CKD stage 5	169 (22.5)	384 (30.4)	3494 (23.0)		138 (21.2)	161 (24.8)	307 (23.6)	
CKD stage 4	186 (24.8)	302 (23.9)	3076 (20.3)		170 (26.2)	164 (25.2)	331 (25.5)	
CKD stage 3	191 (25.5)	320 (25.3)	3739 (24.7)		162 (24.9)	158 (24.3)	323 (24.9)	
CKD stage 1 and 2	202 (27.0)	257 (20.3)			180 (27.7)	167 (25.7)	339 (26.1)	
Antiplatelet	169 (22.5)	295 (23.3)	1781 (11.7)	<0.001	150 (23.1)	138 (21.2)	267 (20.5)	0.433
Statin	304 (40.6)	508 (40.2)	4502 (29.7)	<0.001	271 (41.7)	268 (41.2)	558 (42.9)	0.741

1 Bonferroni correction for continuous variables and the χ^2^ test for categorical variables. *Post hoc* analysis consisted of t-tests with the Bonferroni correction for continuous variables and the χ^2^ test for categorical variables.

ACEi, angiotensin-converting-enzyme inhibitor; ARB, angiotensin receptor blocker, AKD, acute kidney disease; CABG, coronary artery bypass graft; CKD, chronic kidney disease; COPD, chronic obstructive pulmonary disease ; CT, computer tomography; DM, diabetic mellitus; eGFR, estimated glomerular filtration rate; IABP, intra-aortic balloon pump; MV, mechanical ventilator use; NTD, New Taiwan Dollar; PTCA, percutaneous transluminal coronary angioplasty.

Given the differences in baseline characteristics and risk of outcomes of interest between the ACEi/ARB users and nonusers, we matched the three groups using a greedy matching algorithm with a caliper width of 0.2 SDs of the log of the odds of the estimated propensity score with a 1:1:1 ratio. The predictive variables of prescriptions or RASi with logistic regression analysis by applying a propensity score are shown in Supplementary Figure S1. Table 1 shows the covariates adjusted before and after propensity score matching: demographics, age, baseline comorbidities, CKD status, sepsis, medications and severity of kidney function, and the prescription of ACEi/ARB before dialysis initiation.

A forest plot was constructed for the hazard ratio of ACEi vs. ARB users on subsequent mortality according to prior comorbidities and clinical conditions.

All analyses were performed using SAS 9.2 (SAS Institute Inc.) and Stata/MP version 16 (Stata Corporation, TX) for data analysis and figure plotting. A two-sided *p*-value < 0.05 was considered to be statistically significant.

## Results

### Clinical Characteristics of Patients

The study enrolled a total of 41,731 patients who underwent dialysis therapy during their AKI episodes within the study period, of whom 17,141 (41.1%) survival patients could be weaned off from dialysis for at least 7 days during the AKD period. Among them, there were 748 (4.36%) patients with prior ACEi use and 1,263 (7.37%) patients with prior ARB use **(** Figure 1). There were 116 (0.68%) patients with continuing ACEi use and 113 (0.66%) patients with continuing ARB use after weaning from AKI-D. At the AKD period, 100 new users took ARB, and 319 new users took ACEi.

The clinical characteristics of enrolled patients before index admission are shown in Table 1. The indication of RASi usage was mostly attributed to hypertension (*n* = 1711, 85.1%), followed by diabetes (*n* = 507, 25.2%), and prior cerebrovascular accident (266, 13.2%). The ACEi or ARB users before AKI-D had a higher Charlson comorbidity score (2.21 ± 2.58, 3.32 ± 2.57 *vs*.1.48 ± 2.23, *p* < 0.001) than nonusers. The levels of baseline eGFR were significantly lower in prior ACEi or ARB users (44.5 ± 37.7, 38.8 ± 41.5 vs. 50.1 ± 48.1 ml/min/1.73 m^2^, respectively, both *p* < 0.001) than those of the patients without prior ACEi or ARB.

During index hospitalization (Table 2), compared with the patients using ACEi or ARB, nonusers had significantly high ratios of using mechanical ventilation and ICU admission yet had a low ratio of receiving coronary artery bypass graft or percutaneous transluminal coronary angioplasty. The patients who were administered ACEi or ARB also were more likely to take other antihypertensive agents than nonusers at the AKD period. Additionally, compared with nonusers, ACEi and ARB users both had a high ratio of having been prescribed with statins and anti-urate medications after dialysis withdraw.

**TABLE 2:** Comparison of patient characteristics at index admission and clinical outcome among ACEi users, ARB users, and other drug users.

Characteristic	Before match				Propensity score-matched			
	ACEi user (*n* = 748)	ARB user (*n* = 1,263)	Nonuser (*n* = 15,130)	*p*-value	ACEi user (*n* = 650)	ARB user (*n* = 650)	Nonuser (*n* = 1,300)	*p*-value^1^
Hospitalization days				0.790	—	—	—	0.758
≤14 days	221 (29.5)	379 (30.0)	4340 (28.6)		188 (28.9)	191 (29.4)	384 (29.5)	
15–30 days	268 (35.8)	452 (35.7)	5614 (37.1)		237 (36.5)	234 (36.0)	497 (38.2)	
≥30 days	259 (34.6)	432 (34.2)	5176 (34.2)		225 (34.6)	225 (34.6)	419 (32.2)	
Sepsis	318 (42.5)	585 (46.3)	6945 (45.9)	0.178	282 (43.4)	311 (47.9)	567 (43.6)	0.159
Intervention during hospitalization								
Mechanical ventilation ≥4 days	330 (44.1)	547 (43.3)	7178 (47.4)	0.005	287 (44.2)	299 (46.0)	562 (43.2)	0.509
Pleural effusion	41 (5.4)	64 (5.0)	690 (4.5)	0.380	37 (5.7)	38 (5.9)	62 (4.8)	0.517
Chest intubation	43 (5.7)	48 (3.8)	743 (4.9)	0.110	38 (5.9)	28 (4.3)	68 (5.2)	0.448
ICU admission	521 (69.6)	898 (71.1)	11104 (73.3)	0.021	461 (70.9)	480 (73.9)	928 (71.4)	0.428
MV	691 (92.3)	1177 (93.1)	14105 (93.2)	0.670	604 (92.9)	604 (92.9)	1205 (92.7)	0.974
CABG	17 (2.2)	34 (2.6)	265 (1.7)	0.039	15 (2.3)	17 (2.6)	26 (2.0)	0.678
PTCA	52 (6.9)	101 (8.0)	887 (5.8)	0.006	46 (7.1)	50 (7.7)	96 (7.4)	0.914
IABP	18 (2.4)	34 (2.6)	453 (2.9)	0.557	18 (2.8)	16 (2.5)	42 (3.2)	0.613
CT	167 (22.3)	322 (25.4)	3958 (26.1)	0.061	149 (22.9)	179 (27.5)	345 (26.5)	0.123
Medication in the index hospitalization but before weaning								
Anti-uric acid	136 (18.1)	203 (16.0)	2238 (14.7)	0.023	105 (16.2)	104 (16.0)	194 (14.9)	0.716
Antiplatelet drug	262 (35.0)	458 (36.2)	5332 (35.2)	0.756	232 (35.7)	242 (37.2)	473 (36.4)	0.846
Statin	129 (17.2)	214 (16.9)	2059 (13.6)	<0.001	112 (17.2)	108 (16.6)	254 (19.5)	0.216
Antihypertensive drugs								
alpha-blocker	14 (1.9)	31 (2.5)	153 (1.0)	<0.001	12 (1.9)	17 (2.6)	15 (1.2)	0.058
Beta-blocker	110 (14.7)	179 (14.2)	968 (6.4)	<0.001	92 (14.2)	91 (14.0)	104 (8.0)	<0.001
Ca-blocker	126 (16.8)	246 (19.5)	1546 (10.2)	<0.001	114 (17.5)	125 (19.2)	146 (11.2)	<0.001
Diuretics	16 (2.1)	49 (3.9)	230 (1.5)	<0.001	14 (2.2)	24 (3.7)	25 (1.9)	0.049
Others	25 (3.3)	52 (4.1)	306 (2.0)	<0.001	22 (3.4)	21 (3.2)	32 (2.5)	<0.001
eGFR at AKD	27.7 ± 30.1	27.2 ± 27.5	34.9 ± 40.5	<0.001	27.5 ± 29.1	29.7 ± 28.9	28.6 ± 29.7	0.429
Outcomes of interests								
3M-death	204 (27.2)	316 (25.0)	4007 (26.4)	0.453	178 (27.4)	157 (24.1)	343 (26.4)	0.389
6M-death	261 (34.8)	398 (31.5)	4853 (32.0)	0.240	231 (35.5)	205 (31.5)	427 (32.9)	0.288
1Y-death	316 (42.2)	506 (40.0)	5634 (37.2)	0.004	284 (43.7)	250 (38.5)	504 (38.8)	0.076
All-cause death	366 (48.9)	659 (52.1)	6261 (41.3)	<0.001	334 (51.4)	315 (48.5)	585 (45.0)	0.024
Re-dialysis	304 (40.6)	536 (42.4)	4938 (32.6)	<0.001	274 (42.2)	240 (36.9)	340 (39.5)	0.077

1 Bonferroni correction for continuous variables and the χ2 test for categorical variables. *Post hoc* analysis consisted of t-tests with the Bonferroni correction for continuous variables and the χ2 test for categorical variables.

ACEi, angiotensin-converting-enzyme inhibitor; ARB, angiotensin receptor blocker, AKD, acute kidney disease; CABG, coronary artery bypass graft; CKD, chronic kidney disease; COPD, chronic obstructive pulmonary disease ; CT, computer tomography; DM, diabetic mellitus; eGFR, estimated glomerular filtration rate; IABP, intra-aortic balloon pump; MV, mechanical ventilator use; NTD, New Taiwan Dollar; PTCA, percutaneous transluminal coronary angioplasty.

### Risk of Mortality in ACEi Users, ARB Users, and ACEi/ARB Nonusers Among AKI-D Patients

The enrollees were divided into three groups: ACEi users (*n* = 650), ARB users (*n*= 650), and non-RASi users (*n* = 1,300) after propensity score matching (Figure 1; Supplementary Figure S1).

**FIGURE 2:**
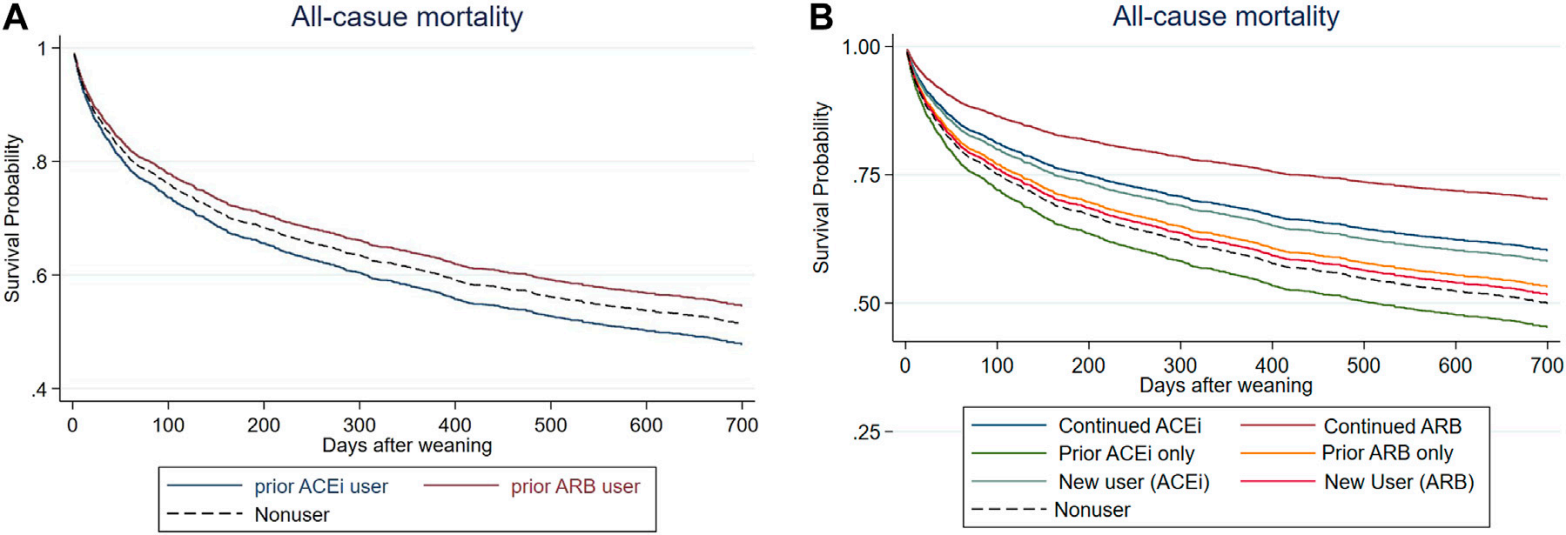
Cox proportional plots depicting **(A)** survival probability levels of before-dialysis ARB and ACEi users, as well as nonusers, and **(B)** survival probability levels of prior ARB, prior ACEi, continuing ARB, continuing ACEi, and new users, as well as nonusers.

After a mean follow-up period of 1.01 ± 0.94 years (Figure 2A and Table 3), it was found that the patients who received ARB had a lower risk of all-cause mortality for post-AKI-D (adjusted hazard ratio (HR); 95% confidence interval (CI): 0.88; 0.77–1.00, *p* = 0.038) than non-RASi users by Cox proportional hazard analysis.

**TABLE 3:** Outcomes associated with prior use and continued use of ACEi or ARB in patients weaning from dialysis requiring AKI.

	Crude HR (*n* = 17141)	Confounder adjusted HR (*n* = 17141)	PS-matched HR (*n* = 2600)
90-days mortality			
ACEi, continued	0.16 (0.07–0.35)***	0.18 (0.08–0.40)***	0.20 (0.09–0.44)***
ARB, continued	0.16 (0.07–0.37)***	0.19 (0.09–0.43)***	0.20 (0.06–0.62)**
ACEi, prior	1.16 (1.01–1.34)*	1.09 (0.95–1.26)	1.09 (0.90–1.31)
ARB, prior	0.96 (0.85–1.07)	0.83 (0.74–0.94)**	0.83 (0.68–1.02)
New-users ACEi	0.27 (0.19–0.39) ***	0.29 (0.20–0.42)***	0.13 (0.02–0.93)*
New-users ARB	0.03 (0.00–0.22) ***	0.04 (0.01–0.30)**	0.00 (0.00–0.00)
180-days mortality			
ACEi, continued	0.38 (0.24–0.60)***	0.42 (0.26–0.66)***	0.62 (0.36–1.09)
ARB, continued	0.26 (0.15–0.46)***	0.29 (0.17–0.52)***	0.31 (0.14–0.69)**
ACEi, prior	1.19 (1.04–1.35)**	1.11 (0.97–1.26)	1.10 (0.93–1.30)
ARB, prior	0.97 (0.87–1.08)	0.83 (0.74–0.92)**	0.87 (0.73–1.03)
New-users ACEi	0.44 (0.34–0.57) ***	0.46 (0.36–0.61)***	0.19 (0.05–0.78)*
New-users ARB	0.19 (0.10–0.39) ***	0.26 (0.13–0.52)***	0.29 (0.04–2.05)
Long-term mortality			
ACEi, continued	0.68 (0.50–0.91)*	0.71 (0.53–0.96)*	0.73 (0.53–1.31)
ARB, continued	0.54 (0.39–0.75)***	0.56 (0.41–0.77)***	0.51 (0.30–0.87)*
ACEi, prior	1.20 (1.08–1.35)**	1.12 (1.00–1.25)	1.14 (0.99–1.32)
ARB, prior	1.05 (0.97–1.15)	0.89 (0.81–0.97)**	0.91 (0.79–1.05)
New-users ACEi	0.70 (0.58–0.84)***	0.74 (0.61–0.88)**	0.78 (0.43–1.43)
New-users ARB	0.36 (0.24–0.56)***	0.46 (0.30–0.70)***	0.95 (0.39–2.31)
Re-dialysis^1^			
ACEi, continued	1.42 (1.10–1.84)**	1.21 (0.95–1.54)	1.26 (0.94–1.67)
ARB, continued	1.55 (1.21–1.98)**	1.15 (0.90–1.47)	1.13 (0.76–1.66)
ACEi, prior	1.25 (1.10–1.42)**	1.10 (0.97–1.25)	1.13 (0.96–1.33)
ARB, prior	1.20 (1.09–1.31)***	0.86 (0.78–0.94)***	0.87 (0.74–1.01)
New-users ACEi	1.55 (1.33–1.81)***	1.34 (1.15–1.56)***	2.22 (1.55–3.18) ***
New-users ARB	1.50 (1.13–1.98)**	1.02 (0.78–1.33)	1.01 (0.53–1.92)

*p < 0.05; **p < 0.01; ***p < 0.001.

1 Taking mortality as a competing risk factor and expressed as a sub-distribution hazard ratio.

ACEi, angiotensin-converting-enzyme inhibitor; ARB, angiotensin receptor blocker; PS, propensity score matching.

The patients were further grouped according to the status of prior (before index dialysis) or continuing (both before and at the AKD period) RASi use to identify the effect of respective RASi. In a further analysis, compared with non-RASi users, patients who received continuing ARB after AKI-D had a significantly low risk for post-weaning all-cause mortality (adjusted HR 0.51; 95% CI: 0.30–0.87, *p* = 0.013) (Figure 2B; Table 3). The beneficial HR for lower mortality in continuing ARB users showed persistent attenuation in a latency time-dependent manner from 90-days (HR, 0.20), 180-days (HR, 0.31), even to a mean follow-up of 1.23 (±1.06) years (HR, 0.51).

### Hospitalization for MACE and MACE-Related Death Among ACEi/ARB Users

Regarding hospitalization for MACE, we did not identify a statistically significant protective effect for ARB relative to ACEi (adjusted HR 0.86; *p* = 0.145). The data did not present a statistically significant protective effect for ARB regarding MACE-related death, either (adjusted HR 0.83; *p* = 0.537).

### Risk of Long-Term ESKD in Prior Users, Continuing Users, and Nonusers of ACEi/ARB

**FIGURE 3:**
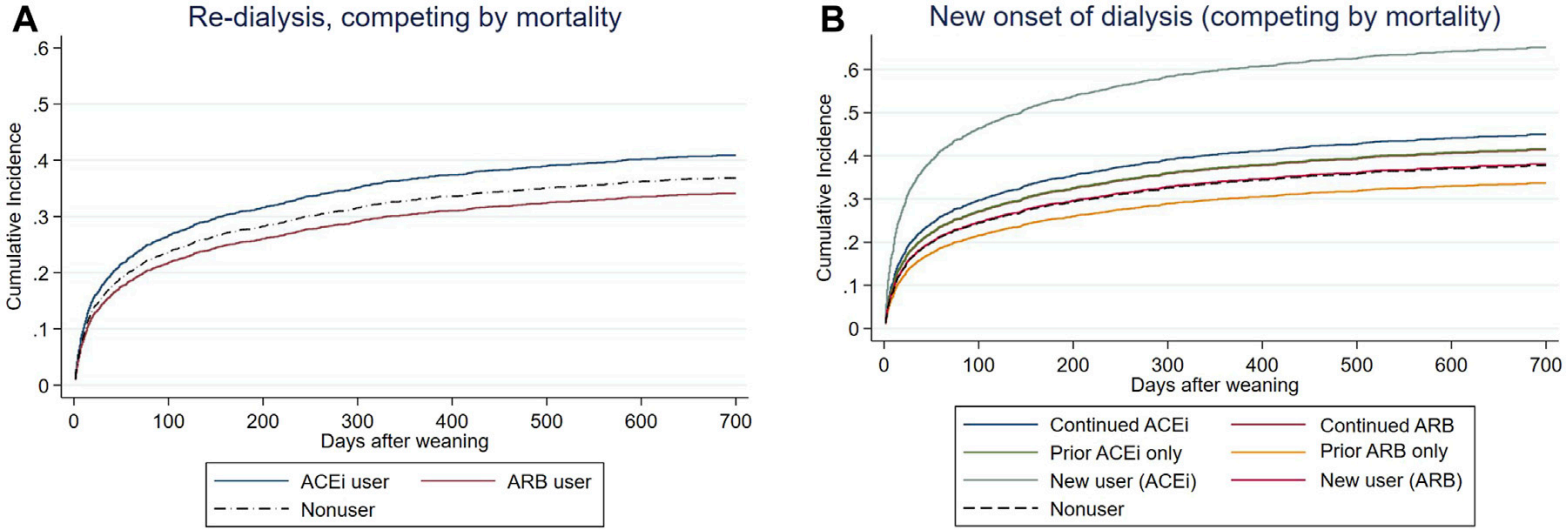
Cox proportional plots depicting **(A)** end-stage kidney disease (ESKD) risk levels of before-dialysis ARB and ACEi users, as well as nonusers, and **(B)** ESKD risk levels of prior ARB, prior ACEi, continuing ARB, continuing ACEi, and new users, as well as nonusers, taking mortality as a competing risk after AKI-D.

Prior ACEi users and prior ARB users did not have increased risk of subsequent ESKD after using mortality as a competing risk factor compared to non-RASi users (Figure 3A, Supplementary Table S1). The patients who had continuing ACEi therapy after AKI-D could not decrease the risk of all-cause mortality and subsequent ESKD compared with the RASi nonusers even after adjusting kidney function at AKD and taking mortality as a competing risk factor (Table 3; Figure 3B).

In the other compatible groups, those patients who had only prior/continuing ARB usage or only prior ACEi usage did not have increased risk for re-dialysis following weaning from AKI-D in this matched COX proportional hazard model, taking mortality as a competing risk. However, the new users of ACEi at the AKD period had increased risk of re-dialysis (adjusted sub-distribution hazard ratio (sHR) 2.22; 95% CI: 1.55–3.18, *p* = 0.037).

### Sensitivity Analysis of ARB vs. ACEi

**FIGURE 4:**
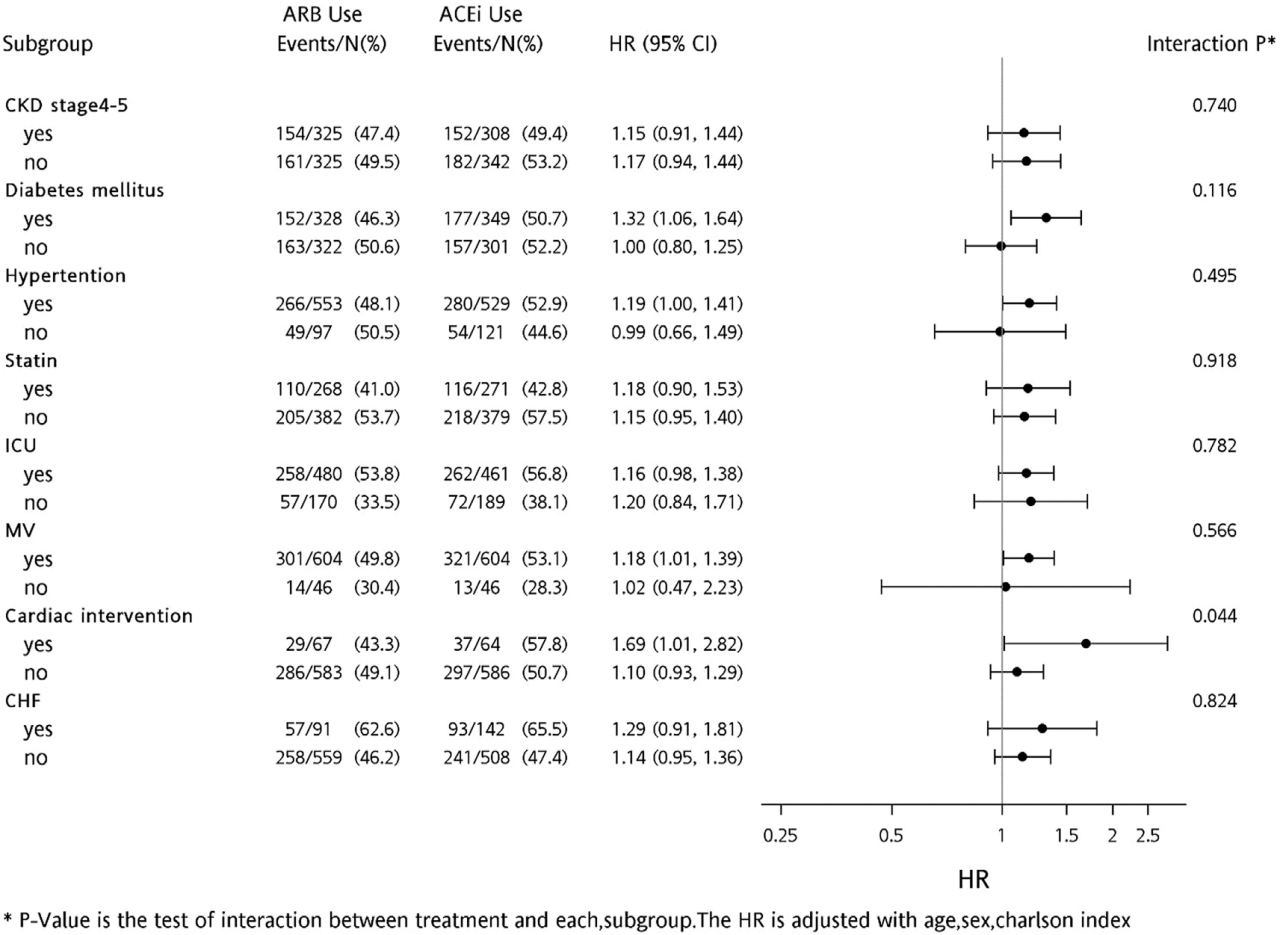
Forest plot comparing risk levels of all-cause mortality for before-dialysis ACEi and ARB users.

In regard to all-cause of mortality, pre-dialysis ARB users had a lower rate than ACEi users (HR, 0.824; *p* = 0.017). To compare differences between ACEi and ARB, we identified respective findings in the subgroup analysis (Figure 4). In patients with prior hypertension, diabetes, mechanical ventilation use, and cardiac intervention, risks of mortality were significantly lower in those taking ARB than those taking ACEi, before dialysis initiation.

### Complication Analysis

We further analyzed the episodes of hyperkalemia, defined by serum potassium greater than 5.3 mmol/L (upper limit of the normal range), and found that continued use of both ARB (*p* = 0.070) and ACEi (*p* = 0.219) did not increase the risk of hyperkalemia after weaning from AKI-D. For each patient, we also collected data on eGFR in the 1 year following withdrawal of dialysis and calculated the mean level. The mean eGFR values for ARB user, ACEi users, and nonusers are 32.7, 28.7, and 30.6, respectively. No statistically significant difference was detected (*p* = 0.103).

### Negative Analysis

To attribute the possible health indication biases or unobserved confounders, we further identified the risk of new onset of gastrointestinal bleeding after ACEi or ARB usage. The patients with continued ACEi usage (*p* = 0.198) or ARB usage (*p* = 0.157) had similar risks of gastrointestinal bleeding after weaning from AKI-D.

## Discussion

This study is the first large population-based cohort study to investigate the role of ACEi versus ARB, respectively, in the long-term risks of mortality and re-dialysis in AKD patients weaned-off from AKI-D. Nearly two-fifths of AKI-D patients could be weaned off from dialysis for at least 7 days. We showed that patients weaning from AKI-D and had pre-AKI-D-continued ARB usage were associated with lower risk of long-term all-cause mortality. Further analysis showed the benefit of ARB mainly stemmed from continuing ARB usage, but those who had continuing ACEi usage did not decrease their mortality risk. During a mean follow-up period of 1.01 years after being weaned-off from AKI-D, patients with *de novo* ACEi usage had a higher risk of re-dialysis than those without ACEi/ARB usage, even after adjustment of their kidney function recorded at AKD. We also demonstrated that there was not an increased risk of hyperkalemia associated with using ACEi/ARB after weaning from AKI-D.

### ACEi and ARB Are Different in Clinical Scenarios

In the real-world practice, discontinuation of ACEi/ARB during admission and/or acute illness is common, particularly in patients with AKI-D. Both ACEi and ARB are RASi and have traditionally been considered to have similar clinical effects. Indeed, when treating patients with hypertension, heart failure, diabetes, cardiovascular disease, and chronic kidney disease, head-to-head studies revealed the antihypertensive efficacy and various clinical outcomes between ACEi and ARB were comparable (15). However, our current study provided the first evidence that their effects on reducing the risk of all-cause mortality after weaning from AKI-D may be different at the AKI to CKD transition.

We found that AKD patients who had continuing ARB usage had lower risk of all-cause mortality, whereas there was no increased risk of re-dialysis after AKI-D/AKD. However, the mortality rate in continuing ACEi users was not remarkably lower than that in the non-RASi users. Since the transition from AKI to AKD and to CKD is an interconnected syndrome, our study provides a novel insight into the feasibility of various pharmacological therapies influencing the post-AKI care and even the prognosis of these patients (Ostermann et al., 2017).

In some clinical scenarios, ARB could provide better protection than ACEi does. For example, in the REACH cohort, a real-world practice, ARB was superior to ACEi in reducing cardiovascular events among high-risk patients (Potier et al., 2017); moreover, in patients who underwent coronary artery bypass grafting (CABG), the incidence of major cardiovascular events was significantly lower in ARB users during their 12-month follow-up period (Kim et al., 2020). In addition to cardiovascular protection, ARB was associated with lower rates of sepsis than ACEi in patients with chronic obstructive pulmonary disease (Lai et al., 2019); furthermore, patients who were administered ARB, rather than ACEi, had lower rates of hospitalization for sepsis, than untreated patients (Dial et al., 2014).

According to a secondary analysis of the RENAL study in critically ill patients with AKI, ACEi administration during the follow-up period was infrequent and was not associated with the statistically significant impact upon patient survival (Wang et al., 2014). A previous study found that the use of RASi did not show higher rates of ESKD possibly because the deteriorated effects of ACEi could be ameliorated by ARB use (Brar et al., 2018). However, in this study, we noticed higher risk of ESKD, as a solid outcome of kidney events in patients who were added with either *de novo* RAS inhibitors when they were weaned-off from AKI-D.

### ACEi and ARB Are Different in Pharmacological Mechanisms

In patients administered with ARB or ACEi, plasma angiotensin II levels were augmented in the ARB group but no similar findings were noticed in the ACEi group (Nakamura et al., 2009); thus, ARB might facilitate the effects of angiotensin II type 2 receptor (AT2R)-mediated responses to the increased levels of angiotensin II. The anti-inflammatory effect of ARB could be more potent than that of ACEis; for example, ramipril, an ACEi, increases IL-1β and IL-10 in patients with kidney diseases (Gamboa et al., 2012). ACEi increases the plasma levels of asymmetric dimethylarginine (ADMA), an endogenous inhibitor of nitric oxide synthases as compared with valsartan, an ARB (Gamboa et al., 2015).

ACE2 expression is abundant in the kidney and is thought to provide protection against kidney injury (Williams and Scholey, 2018). Preclinical analysis showed inconsistent findings regarding the effects of RASi on ACE2 expression (Ferrario et al., 2005). ACEi could decrease (Hamming et al., 2008) or did not affect the activity of ACE2 (Rice et al., 2004), while ARB has been shown to increase urinary ACE2 secretion in hypertensive patients, which indicates that upregulation of ACE2 may be present in humans (Furuhashi et al., 2015). ARB could play a key role by increasing expressions of ACE2 and plasma angiotensin-(1–7) in animals and humans, thus modifying processes associated with acute inflammation and inhibiting leukocyte activation and recruitment (Simoes e Silva et al., 2013). Increased levels of angiotensin II occurring after ARB treatment, but not after ACEi, would increase substrate load on ACE2, thus leading to its upregulation (Esler and Esler, 2020). In light of our findings, continuing (reinitiating) ARB use, but neither adding new ACEi or ARB in RASi-naïve patients, nor continuing (re-initiating) ACEi use after AKI-D patients weaned off from AKI-D, might be helpful to decrease subsequent all-cause mortality.

### Association of Use of ACEi and ARB With Hyperkalemia

Hyperkalemia is a possible caveat of the RASi prescription. However, we found that patients given either ARB or ACEi did not have increasing risk of hyperkalemia following the AKI-D.

### Study Strengths and Limitations

It is the first large-scale study to investigate the individual impact of ACEi versus ARB in patients after weaning from AKI-D via utilization of a well-maintained high-quality population cohort, in which the selection bias could be reasonably minimized. Additionally, we had a longer follow-up period than that was usually reported in clinical trials and thus enabled the evaluation of longer-term risks and benefits of RASi therapy in the real-world practice.

Our study also has some limitations. First, our study was an observational study; therefore, the associations were not prospective, and strong causality cannot be inferred. Some important covariates, such as blood pressure, urine output, and body mass index after discharge were not available in our cohort. The observational nature of this study was an intrinsic and unavoidable limitation because the lack of randomization precluded a definite investigation of treatment advantages. Second, obviously, the treating physicians at the time of prescribing the medication had already made a risk assessment and decided that the benefits of ACEi or ARB outweighed the potential nephrotoxicity. Some patients could present AKI related to RAS inhibitors. However, we found that patients continuing use of ACEis or ARBs among AKD patients did not have increasing risk of re-dialysis. Although eGFR dip has strong association with subsequent progression to end-stage kidney disease (Khan et al., 2022), the current guideline illustrated “permissive kidney injury,” in term of less than 30% increase in creatinine after initial use of ACEi or ARB (Kidney Disease, 2020).

However, it is very challenging to perform a randomized controlled trial because ACEi and ARB are standard therapeutic agents and widely used in treating hypertension, diabetic kidney disease, and congestive heart failure. Moreover, using the validated outcome of gastrointestinal bleeding that was not interfered by ACEi/ARB, we could confirm that the selection bias was minimal, if any, from our study design. Additionally, the disease severity score after propensity score matching is similar between the study groups. Actually, regarding kidney function at baseline and the AKD period after weaning, ARB users in this study had the lowest eGFR than others before matching. Even though the ratios of comorbidities (diabetes, cerebrovascular disease, and myocardial infarction) were more severe in ARB users in our cohort, we still noticed the significant survival benefit of ARB usage.

### Prospective

Given the better outcome efficacy but fewer adverse events with ARB (Messerli et al., 2018), risk-to-benefit analysis in aggregate indicates that at present there is enough evidence to support that prior or continuing ARB usage for managing the patients who could be weaned off from AKI-D. Our findings support the hypothesis that ARB may stabilize the kidney outcomes and reduce mortality among patients who were weaned-off from AKI-D, and thus suggest the use of ARB even in the advanced stage of CKD.

In the future, prospective head-to-head comparison trials are the only ironclad way to compare the efficacy and safety of ARB objectively and to test whether the ARB outcome “paradox” really holds true ironclad.

## Conclusion

In conclusion, our present study revealed that prior and continuing ARB usage was associated with lower risk of mortality after AKI-D patients weaned off dialysis, while the use of ACEi did not have survival benefit. New users of ACEi among these AKD patients had a higher risk of re-dialysis after mortality was controlled as a competing risk. The use of ACEi or ARB at the AKD period did not increase the risk of hyperkalemia. Further prospective randomized studies are needed to verify our findings.

## Data Availability

The data for this study are not publicly available. To protect privacy, the Taiwanese government only allows researchers to analyze data from the Health and Welfare Data Science Center in selected computer rooms of the center. No individual data can be brought outside the center, and researchers can only bring away aggregate statistical results based on raw data. To use the data, a researcher has to apply for permission of using data within the center. To apply for such permission, a researcher has to submit an IRB approval concerning the data use, and has to pay for using data. The amount of payment depends on the volume of data used. Requests to access these datasets should be directed to https://www.nhi.gov.tw/Content_List.aspxcn=468220093AFEB6DF8topn=787128DAD5F71B1A.

## Supplementary Material

Supplementary Table S1 Outcomes associated with previous dialysis usage of ACEi or ARB in patients who could wean from dialysis requiring AKI.

Supplementary Figure S1 Standardized differences of baseline covariates before and after the 1:1 propensity score matching in ACEi vs. ARB user **(A)** and ACEi/ARB vs. other HTN drug-user **(B)**.

## Abbreviations

ACEis, angiotensin-converting-enzyme inhibitors; ACE2, angiotensin-converting enzyme 2; AKD, acute kidney disease; AKI-D, dialysis-requiring acute kidney injury; ARB, angiotensin receptor blocker; CKD, chronic kidney disease; DM, diabetic mellitus; eGFR, estimated glomerular filtration rate; ESKD, end-stage kidney disease; RAS, renin–angiotensin–aldosterone system.
