# Supplementary material for: Angiotensin II Receptor Blocker Associated With Less Outcome Risk in Patients With Acute Kidney Disease
Source: Front Pharmacol. 2022 Apr 20;13:714658. doi: 10.3389/fphar.2022.714658 (PMC9065477; doi:10.3389/fphar.2022.714658)
Supplement: Supplementary file 1 [file Table1.docx]

***Supplementary Table 1***. Outcomes associated with previous dialysis usage of ACEi or ARB in patients who could wean from dialysis requiring AKI.

|  | Crude HR (N=17141) | Confounder  Adjusted HR (N=17141) | PS-Matched HR (N=2600) |
| --- | --- | --- | --- |
| 90-day mortality |  |  |  |
| ACEi# vs others | 1.01 (0.87-1.16) | 0.97 (0.84-1.12) | 0.97 (0.81-1.16) |
| ARB# vs others | 0.90 (0.80-1.01) | 0.80 (0.71-0.90)*** | 0.80 (0.66-0.98)* |
| 180-day mortality |  |  |  |
| ACEi #vs others | 1.06 (0.93-1.20) | 1.01 (0.89-1.15) | 1.01 (0.86-1.19) |
| ARB# vs others | 0.92 (0.83-1.01) | 0.80 (0.72-0.89)*** | 0.84 (0.71-1.00)* |
| Mortality |  |  |  |
| ACEi #vs others | 1.12 (1.01-1.24)* | 1.06 (0.95-1.18) | 1.08 (0.94-1.23) |
| ARB vs others | 1.01 (0.94-1.10) | 0.87 (0.80-0.94)** | 0.88 (0.77-1.00) * |
| Re-dialysis^¶^ |  |  |  |
| ACEi #vs others | 1.26 (1.12-1.41)*** | 1.11 (0.99-1.24) | 1.12 (0.74-1.30) |
| ARB #vs others | 1.21 (1.11-1.32)*** | 0.87 (0.80-0.95)** | 0.86 (0.74-1.00)* |

Abbreviations: ACEi, angiotensin-converting-enzyme inhibitor; ARB, angiotensin receptor blocker; PS, propensity score matching.

*p< 0.05; **p<0.01; ***p<0.001

# mediation prescribed before admission.

^¶^ taking mortality as a competing risk factor.
