# Supplementary material for: Angiotensin II Receptor Blocker Associated With Less Outcome Risk in Patients With Acute Kidney Disease
Source: Front Pharmacol. 2022 Apr 20;13:714658. doi: 10.3389/fphar.2022.714658 (PMC9065477; doi:10.3389/fphar.2022.714658)
Supplement: Supplementary file 2 [file Image1.pdf]

Supplementary Figure 1.

Standardized differences of baseline covariates before and after the 1:1 propensity score matching in ACEi vs. ARB user (A), and ACEi/ARB vs. other HTN drug-user (B)

(A)

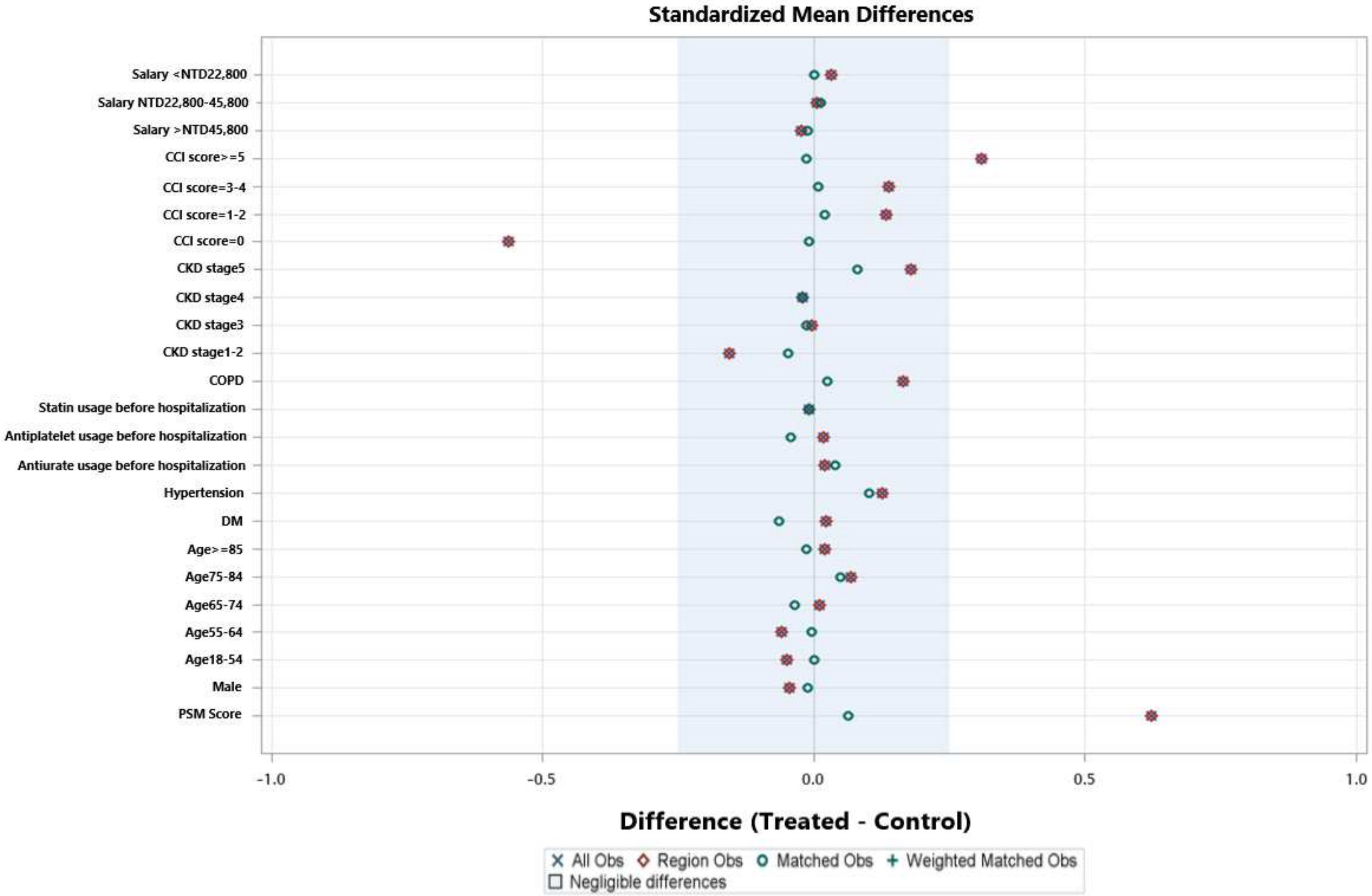

(B)

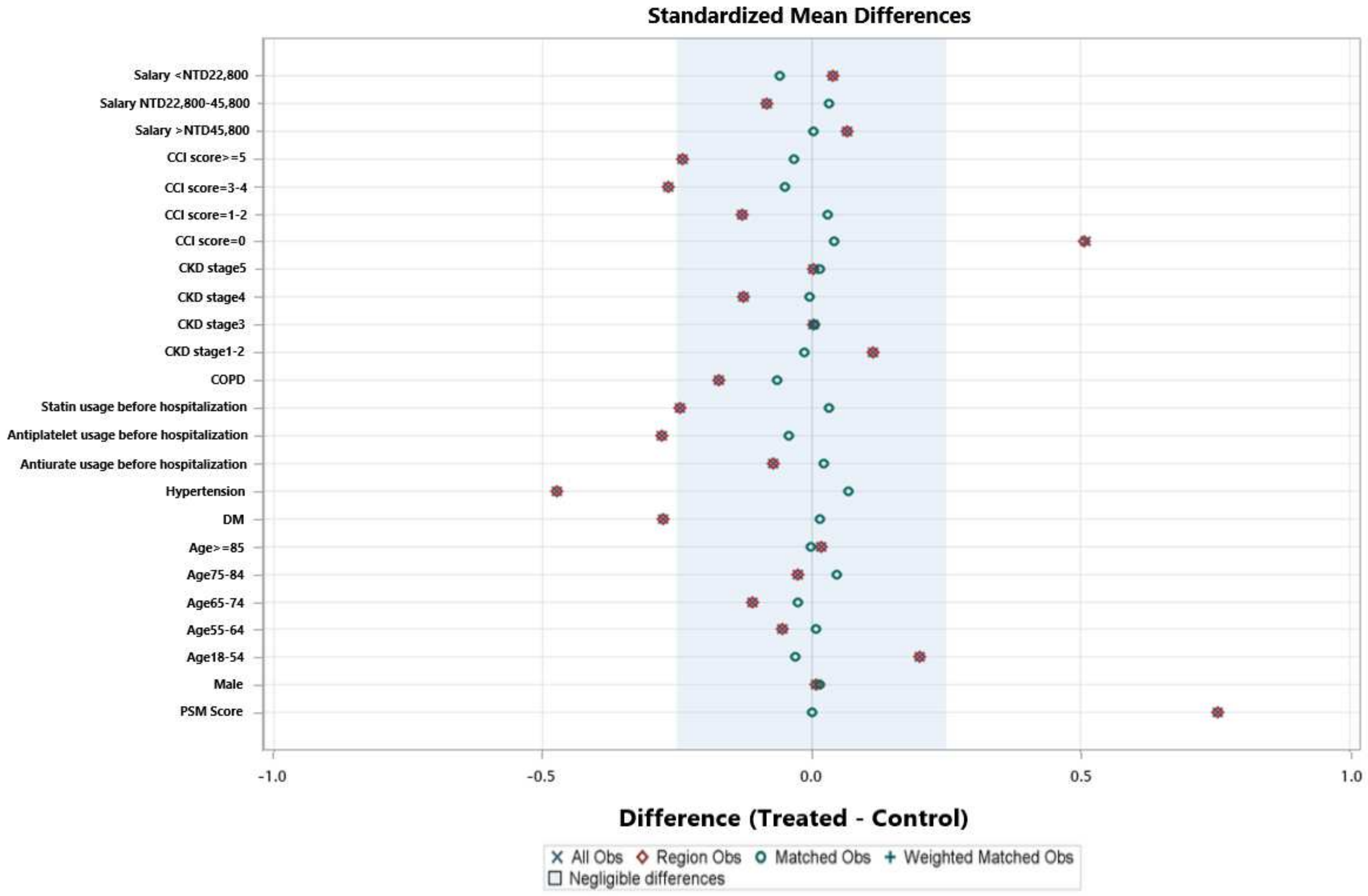

Standardized Mean Differences
Salary <NTD22,800
Salary NTD22,800-45,800
Salary >NTD45,800
CCI score>=5
CCI score=3-4
CCI score=1-2
CCI score=0
CKD stage5
CKD stage4
CKD stage3
CKD stage1-2
COPD
Statin usage before hospitalization
Antiplatelet usage before hospitalization
Antiurate usage before hospitalization
Hypertension
DM
Age>=85
Age75-84
Age65-74
Age55-64
Age18-54
Male
PSM Score
-1.0
-0.5
0.0
0.5
1.0
Difference (Treated - Control)
All Obs
Region Obs
Matched Obs
Weighted Matched Obs
Negligible differences
